# Editorial: Smart endorobots for endoluminal procedures: design, ethics and future trends

**DOI:** 10.3389/frobt.2025.1581829

**Published:** 2025-03-24

**Authors:** Luigi Manfredi, Juan A. Sánchez-Margallo, Helge Arne Wurdemann, Andreas Melzer

**Affiliations:** ^1^ Division of Respiratory Medicine and Gastroenterology, School of Medicine, University Dundee, Dundee, United Kingdom; ^2^ Bioengineering and Health Technologies Unit, Jesus Uson Minimally Invasive Surgery Centre, Cáceres, Spain; ^3^ Department of Mechanical Engineering, University College London, London, United Kingdom; ^4^ Institute and Innovation Center for Computer Assisted Surgery, University Leipzig, Leipzig, Germany

**Keywords:** endorobots, endoscopic robots, robotics navigation, AI, soft robotics, inflammatory bowel disease (IBD)

## 1 Introduction

The development of smart endorobots is transforming the field of minimally invasive procedures by introducing novel solutions for diagnosis and treatment within the human body. These advanced robotic systems enable enhanced manoeuvrability, improved precision, and greater patient safety. Clinicians are increasingly supported by endorobots to perform diagnosis and treatment of difficult-to-reach organs through small incisions or natural orifices. New technologies have provided a tool to improve the design of such devices (e.g., reducing size, improving dexterity, and reducing stress and fatigue), while AI and ML algorithms are providing a tool to support clinical judgment (Manfredi, 2021; Manfredi, 2022).

This new generation of endorobots is not only capable of mimicking users’ movements but also performing tasks at different levels of autonomy. Their design requires a multidisciplinary team that involves experts in various research areas, including actuators, sensors, control, mechanics, Artificial Intelligence (AI) and Machine Learning (ML), and imaging. The increased level of autonomy introduces ethical concerns regarding responsibility, regulatory compliance, and ensuring safety while avoiding limitations on technological advancements.

The design of endorobots for endoluminal applications requires a multidisciplinary approach covering different research aspects. The goal of this Research Topic was to gather contributions from various disciplines, offering readers a comprehensive overview of the field.

## 2 Contents of the Research Topic

This Research Topic includes a collection of articles and reviews that can be summarized into three main themes: i) Navigation, ii) Robotics, and iii) Control and AI. These themes encompass critical advancements in technologies, addressing challenges in manoeuvrability, automation, and medical decision-making.

### 2.1 Navigation

One of the critical challenges in endoluminal robotics is navigation through complex anatomical pathways (Berthet-Rayne and& Yang) introduces Minimal Occupation VolumE (MOVE) a teleoperation method extending the concept of follow-the-leader navigation. This approach optimizes the use of available space surrounding the robot while ensuring safe and efficient movement through tortuous environments. Unlike traditional navigation methods, MOVE provides flexibility and adaptability, addressing constraints such as joint limitations and kinematic complexities. The proposed method has been validated through simulations and control experiments, demonstrating its potential for application in flexible endoscopic surgery.

### 2.2 Robotics

Robotic solutions for endoluminal procedures are advancing towards increased modularity, reusability, and improved adaptability. (Lenssen et al.) presented MISLI-Drive, a modular sterilizable robotic driver for steerable laparoscopic instruments, exemplifies these developments. This robotic driver addresses key challenges in instrument control, sterilization, and user adaptability. It incorporates a quick-release instrument clutching mechanism and embedded sensors for real-time feedback. Compared to existing robotic systems, the MISLI-Drive demonstrates efficient instrument exchange and reduced cleaning complexity, making it a viable solution for sustainable robotic-assisted surgery.

Another contribution is the study on soft robotics and tactile sensing for tissue palpation using Electrical Impedance Tomography (EIT) (Alian et al.). This research integrates soft robotics with EIT-based tactile sensing to enable real-time assessment of tissue stiffness, enhancing the capabilities of flexible endoscopic robots. The findings indicate that soft continuum robots with embedded tactile sensors can improve early-stage cancer diagnostics, providing a non-invasive means to classify tissue abnormalities.

### 2.3 Control and AI

The integration of AI and control strategies is revolutionizing endorobotic systems. A comprehensive review on model-based and model-free control strategies for soft actuators in colonoscopy discusses different control approaches for enhancing robotic manoeuvrability (Asgari et al.). The study emphasizes the importance of adaptive control strategies that can optimize soft robotic movements while minimizing force exerted on the colonic wall. By leveraging AI and sensor-driven feedback loops, these control mechanisms can improve safety and efficiency in endoscopic procedures.

A further contribution to this theme is the research on Self-Supervised Monocular Depth Estimation for High Field of View Colonoscopy Cameras (Mathew et al.). This study presents an AI-based method for estimating depth in colonoscopy images using a self-supervised learning approach. The method enhances robotic navigation by providing 3D spatial awareness in real-time, addressing challenges related to occlusions, low-texture surfaces, and high optical distortion in wide-angle colonoscopy cameras. The integration of such AI-driven depth estimation models holds promise for improving the accuracy and safety of robotic-assisted colonoscopy.

Another article in this Research Topic explores AI applications in the diagnosis and classification of Inflammatory Bowel Disease (IBD) (Braverman-Jaiven and& Manfredi). The study highlights the potential of deep learning algorithms and convolutional neural networks (CNNs) in automating polyp detection and IBD classification. While AI-driven diagnostics have shown promising results, the study underscores the need for larger annotated datasets and more robust validation to enhance reliability and clinical applicability.

## 3 Conclusion

The manuscripts published in this Research Topic showcases significant advancements in smart endorobots for endoluminal procedures, spanning navigation, robotics, and AI-driven control. The contributions highlight the potential of soft robotics, modular robotic drivers, and AI-enhanced control systems to improve surgical precision, reduce patient discomfort, and enhance diagnostic capabilities. However, challenges remain, including the need for regulatory frameworks, integration with existing clinical workflows, and further validation through clinical trials. Future research should focus on refining these technologies to enable widespread adoption in minimally invasive surgery and diagnostic interventions.

The integration of intelligent robotic solutions into endoluminal procedures marks a paradigm shift in the field of medical robotics. By addressing current limitations and harnessing the capabilities of AI, soft robotics, and advanced control systems, the future of minimally invasive interventions is set to become more precise, efficient, and patient-centric.
